# Sirtuin 1 inhibits lipopolysaccharide-induced inflammation in chronic myelogenous leukemia k562 cells through interacting with the Toll-like receptor 4-nuclear factor κ B-reactive oxygen species signaling axis

**DOI:** 10.1186/s12935-020-1152-z

**Published:** 2020-03-06

**Authors:** Lei Wang, Mingming Wang, Hongju Dou, Wenjie Lin, Lifang Zou

**Affiliations:** Department of Hematology, Shanghai Ninth People’s Hospital Affiliated to Shanghai Jiao Tong University School of Medicine, No. 639, Manufacturing Bureau Road, Shanghai, 200011 China

**Keywords:** CML, Inflammation, SIRT1, TLR4, NFκB, ROS

## Abstract

**Background:**

Chronic myelogenous leukemia (CML) is a clonal myeloproliferative neoplasm resulting from BCR–ABL-transformed hematopoietic stem cells. Previous research has implicated multifunctional proinflammatory cytokines in CML development. It has been reported that Sirtuin 1 (SIRT1) as well as ADP-ribosyltransferase and deacetylase may influence CML cell viability and inflammation.

**Methods:**

This study was directed toward exploring the SIRT1-involved in the mechanism of lipopolysaccharide (LPS)-triggered inflammation in CML k562 cells.

**Results:**

In our study, the LPS-induced inflammation in k562 cells was reflected by increases in levels of diverse inflammatory cytokines, including interleukin (IL)-10, IL-1β, IL-6, interferon-γ, tumor necrosis factor (TNF)-α and TNF-β. LPS also decreased SIRT1 expression and nuclear location in k562 cells. Furthermore, SIRT1 overexpression inhibited the release of the above mentioned cytokines in LPS-treated cells. We also determined that LPS stimulation could activate Toll-like receptor 4 (TLR4), the nuclear factor κ B (NFκB) subunit, and p65 and produce reactive oxygen species (ROS) in k562 cells. Nevertheless, SIRT1 overexpression decreased TLR4 expression, thereby repressing the phosphorylation of the NFκB subunit and p65 and decreasing ROS production.

**Conclusions:**

These findings suggest that SIRT1 is a latent therapeutic target for mitigating LPS-induced inflammation via the TLR4–NFκB–ROS signaling axis.

## Background

Chronic myelogenous leukemia (CML) is a clonal hematological malignancy resulting from BCR–ABL-transformed hematopoietic stem cells (HSCs) [1]. There are three clinical phases of CML, progressing from a chronic phase to an accelerated phase and then to a terminal blast crisis. Tyrosine kinase inhibitors (TKIs) target the constitutively activated BCR–ABL kinase, thus leading to a longer-term remission of CML in majority of patients, but they do not eliminate leukemia stem cells (LSCs). Thus, the relapse that occurs in 50% of patients after stopping treatment with TKIs is likely due to the presence of LSCs [2, 3].

Sirtuins (SIRTs) are nicotinamide adenine dinucleotide (NAD)-dependent protein deacetylases that are highly conserved from yeast to mammalian cells. Seven SIRTs (SIRT1–SIRT7) in mammalian cells exhibit functional significance on aging, diabetes, cardiovascular diseases, and cancers [4]. SIRT1, the most extensively studied SIRT, can deacetylate various histone and nonhistone substrates alike including p53, c-MYC, and FOXO, thereby regulating DNA repair, metabolism, cell cycle, and survival [5, 6]. Earlier studies identified tumor suppressor p53 as the first nonhistone SIRT1 deacetylase target: under stress conditions, such as DNA damage, the deacetylation of p53 attenuates its transactivation-dependent apoptosis, thus promoting lung cancer cell survival [7, 8]. Likewise, E2F1 was also found to be negatively regulated by SIRT1 in the lung cancer cell line [9]. Therefore, SIRT1 is considered to be an oncogenic protein.

Cytokines create a proinflammatory environment during CML development, and provide a proliferative advantage to leukemic cells [10, 11]. Several inflammatory cytokines, including granulocyte–macrophage colony-stimulating factor (GM-CSF), interleukin (IL)-6, and IL-1α, are significantly increased during leukemogenesis.; these inflammatory cytokines provide a leukemic environment that might impose malignant cell properties on untransformed cells [11, 12]. Therefore, therapeutic applications targeting the inflammatory environment might restore normal differentiation as well as perturb leukemic cells [13]. Inflammatory signals, sent to respond to environmental stresses, not only can elicit the active cycle of HSCs but also directly trigger these cells to produce cytokines that enhance myeloid differentiation. After stress myelopoiesis is triggered, HSCs inactivate the response through intracellular signaling programs and then return to a stable state. Long-term or immoderate exposure to inflammatory cytokines can result in a continuous cycle and final HSC loss, which enhances bone marrow failure and elicits preleukemic states or leukemia by acquiring genetic and epigenetic variations in HSCs. The phenomena mentioned above can occur by initiating clonal hematopoiesis, with the subsequent appearance of pre-LSCs. In acute myeloid leukemia, LSC cycling and differentiation can be enhanced by activating a few inflammatory signaling pathways [14]. However, the detailed mechanism of inflammation in CML has been rarely reported. In the present study, lipopolysaccharide (LPS) was utilized to trigger inflammation in CML k562 cells and the role of SIRT1 as well as that of the Toll-like receptor 4 (TLR4)–nuclear factor κ B (NFκB)–reactive oxygen species (ROS) signaling axis in inflammation was investigated.

## Materials and methods

### Cell culture

CML k562 cells (Type Culture Collection, Chinese Academy of Sciences, Beijing, China) were cultured in Roswell Park Memorial Institute 1640 medium containing new-born calf serum (10%), streptomycin (100 μg/mL), and penicillin (100 U/mL) at 37 °C in a damp condition (5% CO_2_), followed by exposure to 10 μg/mL of LPS for 4 h.

### Transfection

About 4 × 10^6^ cells were transfected with pCDNA (4 μg), pCDNA-SIRT1 (4 μg), siRNA-NC (8 μg), or siRNA-SIRT1 (8 μg) using the Lipofectamine2000 transfection agent (Thermo Fisher). These cells were used for experiments 36 h after the transfection.

### Western blot (WB)

Cells were subjected to radioimmunoprecipitation assay buffer to prepare lysate. Bicinchoninic acid assay was then used to determine the protein concentration. After resolving via 10% sodium dodecyl sulfate-polyacrylamide gel electrophoresis, proteins were transferred to polyvinylidene difluoride membranes (0.45 µm), which were then blocked with 5% bovine serum albumin (BSA) in phosphate-buffered saline (PBS)-Tween20 (PBS-T) at 25 °C for 60 min and incubated at 4 °C for 12 h with the following immunoglobulins: rabbit anti-IL-1β (1:2000, ab2105, Abcam), anti-IL-6 (1:2000, ab7737, Abcam), anti-TNF-α (1:2000, ab9635, Abcam), mouse anti-SIRT1 (1:1000, ab110304, Abcam), anti-TLR4 (1:2000, ab22048, Abcam), anti-p-NFκB (1:500, ab86299, Abcam), anti-NFκB (1:2000, ab16502, Abcam), and anti-GAPDH (1:5000, ab8245, Abcam). Subsequently, the resulting membranes were incubated with horseradish peroxidase-connected secondary antibodies at 4 °C for another 60 min. Blots were observed through an chemiluminescence reaction via a blot scanner.

### Quantitative polymerase chain reaction (qPCR)

Total RNA was isolated with Trizol as per the manufacturer’s instructions, and messenger RNA (mRNA) levels were measured through qPCR with GAPDH as the internal standard. qPCR was performed using SYBR Green master mix via a real-time PCR system. The PCR reaction was performed at 95 °C for 10 min and cycled 40 times for 15 s and at 60 °C and 72 °C for 30 s each. The 2-ΔΔCT method was used for determining the copy number of objective genes. The primer sequences were as follows: IL-1β F: 5′-CCA CAG ACC TTC CAG GAG AAT G-3′, IL-1β R: 5′-GTG CAG TTC AGT GAT CGT ACA GG-3′; IL-6 F: 5′-AGA CAG CCA CTC ACC TCT TCA G-3′, IL-6 R: 5′-TTC TGC CAG TGC CTC TTT GCT G-3′; TNF-α F: 5′-GAT CTC AAA GAC AAC CAA CAT GTG-3′, TNF-α R: 5′-CTC CAG CTG GAA GAC TCC TCC CAG-3′; IL-10 F: 5′-CAC AAA GCA GCC TTG CAG AA-3′, IL-10 R: 5′-AGA GCA GGC AGC ATA GCA GTG-3′; IFN-γ F: 5′-ATC CCG TGG AGA CTC CTC AA-3′, IFN-γ R: 5′-CCA AAC ACG TAG ACT GGG TAT CC-3′; TNF-β F: 5′-CCC ATG GCA TCC TGA AAC-3′, TNF-β R: 5′-GGA GGC CTG GAA TCC AAT-3′; SIRT1 F: 5′-TGC TGG CCT AAT AGA GTG GCA-3′, SIRT1 R: 5′-CTC AGC GCC ATG GAA AAT GT-3′; TLR4 F: 5′-CCC TGA GGC ATT TAG GCA GCT A-3′, TLR4 R: 5′-AGG TAG AGA GGT GGC TTA GGC T-3′; p65 F: 5′-GGT CCA CGG CGG ACC GGT-3′, TLR4 R: 5′-GAC CCC GAG AAC GTG GTG CGC-3′; GAPDH F: 5′-CAT GGC CTT CCG TGT TCC TA-3′, GAPDH R: 5′-TGT CAT CAT ACT TGG CAG GTT TCT-3′.

### Enzyme-linked immunosorbent assay (ELISA)

IL-1β (BMS224), IL-6 (BMS213), IL-10 (BMS215-2), IFN-γ (KHC4021), TNF-α (BMS223), and TNF-β (BMS202) levels were detected using an ELISA kit from Thermo Fisher in accordance with the manufacturer’s protocol. Absorbance was recorded at OD 543 using a microplate reader POLARstar Omega (BMG Labtech).

### Subcellular fractionation

Cells (1 × 10^6^) were plated on 6 cm dishes and grown for 36 h. Then, the cells were harvested via scraping into a 500 μL cell lysis buffer containing 10 mM HEPES (pH 7.4), 10 mM NaCl, 1 mM KH_2_PO_4_, 5 mM NaHCO_3_, 1 mM CaCl_2_, 0.5 mM MgCl_2_, and 5 mM EDTA with complete protease inhibitor cocktail. Cells were allowed to swell for 5 min, followed by Dounce homogenization for 50-time strokes. Then, the cells were centrifuged at 7500 rpm for 5 min, generating a pellet containing nuclei and debris and a supernatant of cytosol and plasma. Pellets were resuspended in a 1 mL buffer containing 10 mM Tris (pH 7.5), 300 mM sucrose, 1 mM EDTA, and 0.1% NP40 with complete protease inhibitor cocktail, and then pelleted, resuspended, and washed twice. The final pellets were pure nuclei.

### Determination of SIRT1 activity

SIRT1 activity in whole cell lysate was measured using the SIRT1 fluorometric kit (ab156065, Abcam). Briefly, the assays were performed by incubating the whole cell lysate and Fluoro-Substrate Peptide (0.2 mM), Developer, and NAD (2 mM) at 37 °C for 30 min. Fluorescent intensity was measured on a microplate reader POLARstar Omega (BMG Labtech) with excitation set at an Ex/Em = of 350/460 nm.

### ROS determination

ROS production in k562 cells was determined using dichloro-dihydro-fluorescein diacetate (DCFH-DA), a fluorescence probe. Following the LPS treatment for 4 h, cells were incubated with DCFH-DA (10 μM) away from light at 37 °C for 30 min. A fluorescence microscope was used to determine fluorescence intensity (Ex 488/Em 525 nm) using a microplate reader POLARstar Omega (BMG Labtech). For NAC treatment, 5 μM NAC was used to treat k562 cells for 4 h.

### Immunofluorescence assay (IFA)

IFAs were performed using the Leica SP8 confocal laser scanning microscope. Coverslips carrying k562 cells were washed with PBST (PBS plus 1% Triton X-100) and then fixed with 4% paraformaldehyde. Cells were then blocked with 4% BSA dissolved in PBST and stained with anti-SIRT1 antibody. The secondary antibodies were tetramethyl rhodamine isocyanate-conjugated anti-rabbit IgG.

### Statistical analysis

Data were expressed as mean ± standard deviation. An analysis of variance or t-test was applied to analyze significant differences in variables among the groups. A P-value of < 0.05 indicated significance.

## Results

### LPS induced inflammation in k562 cells

**Fig. 1:**
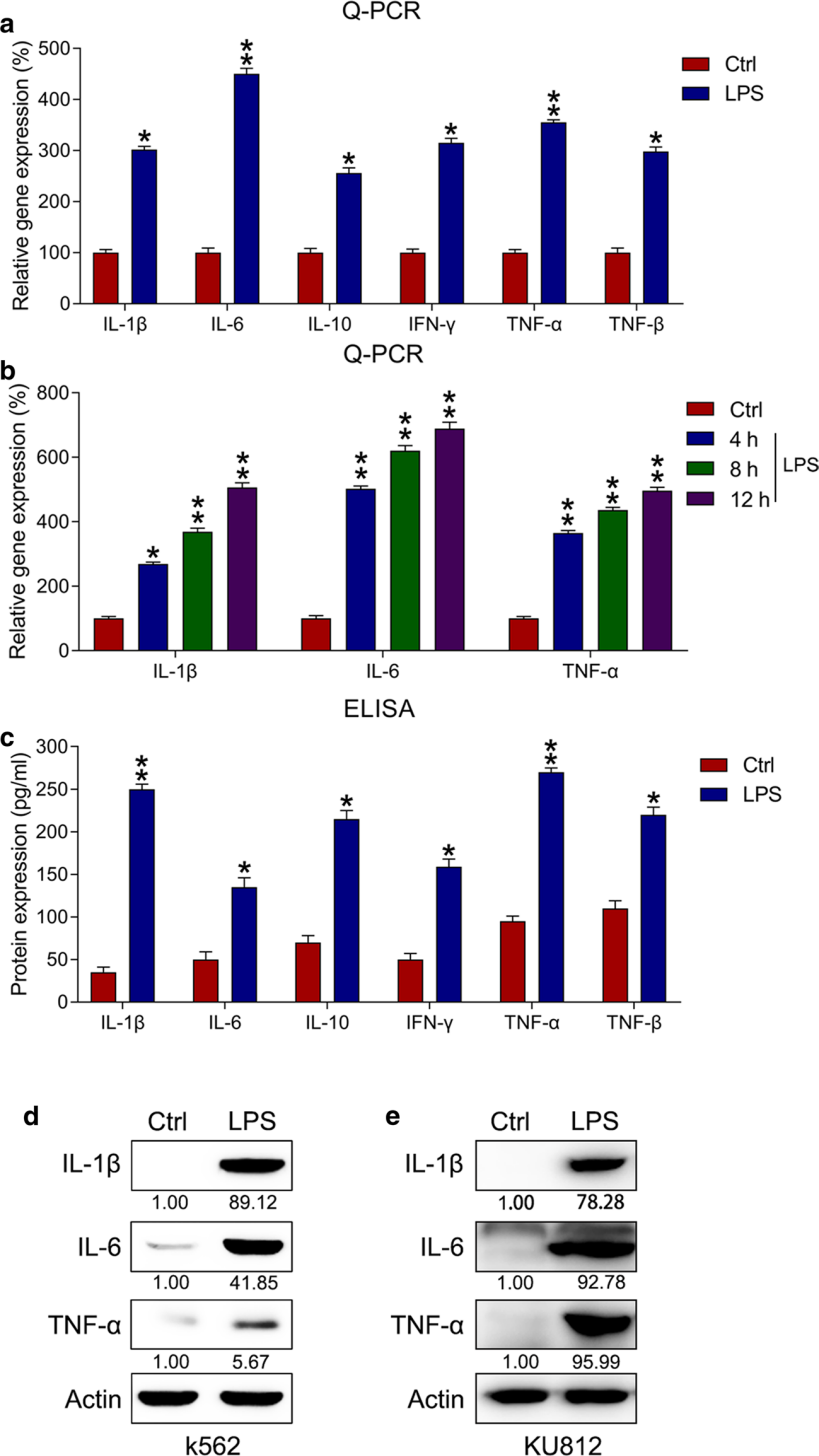
LPS-triggered production of proinflammatory cytokines in k562 cells. Cytokines and chemokines in LPS-treated k562 cells were detected using qPCR, ELISA, and WB. **a** Influence of LPS treatment on mRNA expressions of inflammatory cytokines in k562 cells determined via qPCR. **b** qPCR was performed to examine the mRNA expression of inflammatory cytokines after LPS treatment in k562 cells. In qPCR, gene expressionin each group was first normalized to the GAPDH gene, and then normalized to the data of the control group. **c** Influence of LPS treatment on the protein expressions of inflammatory cytokines in k562 cells determined via ELISA. **d**, **e** Influence of LPS treatment on protein levels of IL-1β, IL-6, and TNF-α in both k562 and KU812 cells determined via WB analyses. Data are expressed as mean ± standard deviation. *P < 0.05, **P < 0.01 relative to the control

First, we examined the proinflammatory role of LPS in k562 cells. These cells were subjected to 10 μg/mL of LPS for 4 h. Subsequently, ELISA and qPCR were used to assess the levels of diverse proinflammatory cytokines. LPS treatment markedly increased in the mRNA levels of inflammatory cytokines in k562 cells (Fig. 1a). Furthermore, a time course experiment was performed to examine IL-1β, IL-6, and TNF-α mRNA levels at 4, 8, and 12 h after the LPS stimulation in k562 cells. The production of IL-1β, IL-6, and TNF-α mRNA gradually increased at 4–12 h posttreatment (Fig. 1b); meanwhile, their protein levels were likewise obviously increased in k562 cells (Fig. 1c). Moreover, IL-1β, IL-6, and TNF-α levels were elevated in both k562 and KU812 cells compared with those in the control group (Fig. 1d, e).

### SIRT1 expression is reduced after LPS treatment

**Fig. 2:**
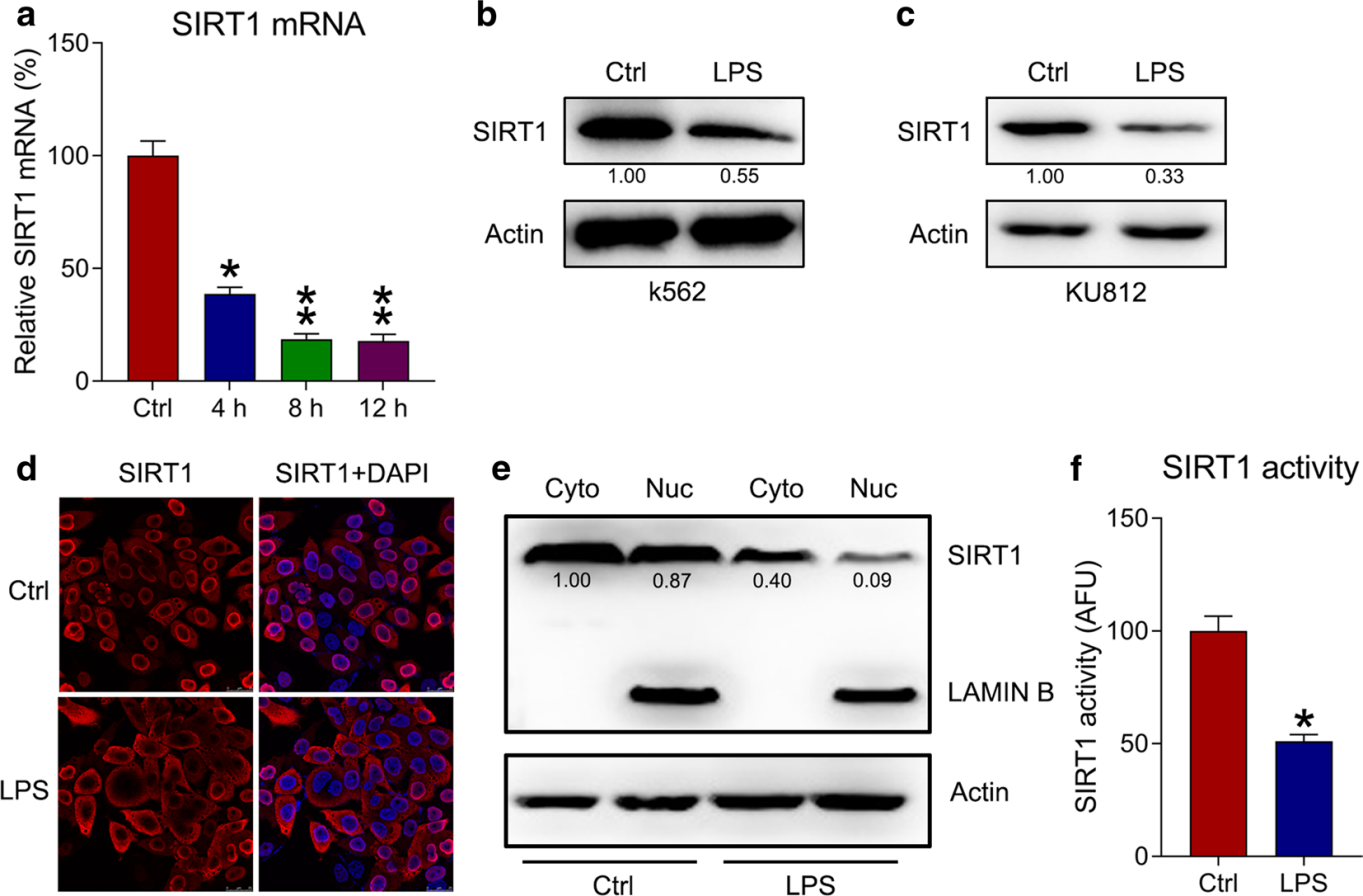
SIRT1 expression in LPS-treated k562 cells. **a** qPCR was used to measure the mRNA expression of SIRT1 at 0, 4, 8, and 12 h after LPS treatment in k562 cells. **b**, **c** WB was performed to detect SIRT1 protein levels in k562 and KU812 cells after LPS treatment. **d** SIRT1 subcellular localization in LPS-treated k562 cells detected using IFA. SIRT1 was stained to red using anti-SIRT1 antibody (red), whereas nuclear DNA was counterstained blue using DAPI (blue). The merged image displays the nuclear localization of SIRT1. **e** Cell fractionation assay showed the location of SIRT1 in nuclear and cytoplasmic fractions. **f** SIRT1 activity was detected in the whole cell lysate. Data are expressed as mean ± standard deviation. *P < 0.05, **P < 0.01 relative to the control

Recently, different roles of SIRT1 in several hematologic malignancy subtypes have been reported [15]. In the present study, k562 cells were treated with LPS and qPCR was used to detect mRNA levels of SIRT1 at different time points; the levels were found to be lower in the LPS group than in the control group in time dependent manner (Fig. 2a). WB analyses showed that LPS treatment reduced SIRT1 expression in both k562 and KU812 cells (Fig. 2b, c). Meanwhile, IFA and cell fractionation showed that LPS treatment reduced the nuclear location of SIRT1 in k562 cells (Fig. 2d, e). In addition, SIRT1 in the whole cell lysate was significantly decreased (Fig. 2f). These findings indicate that SIRT1 is downregulated and deactivated during inflammation.

### Influence of SIRT1 overexpression on LPS-triggered inflammation in k562 cells

**Fig. 3:**
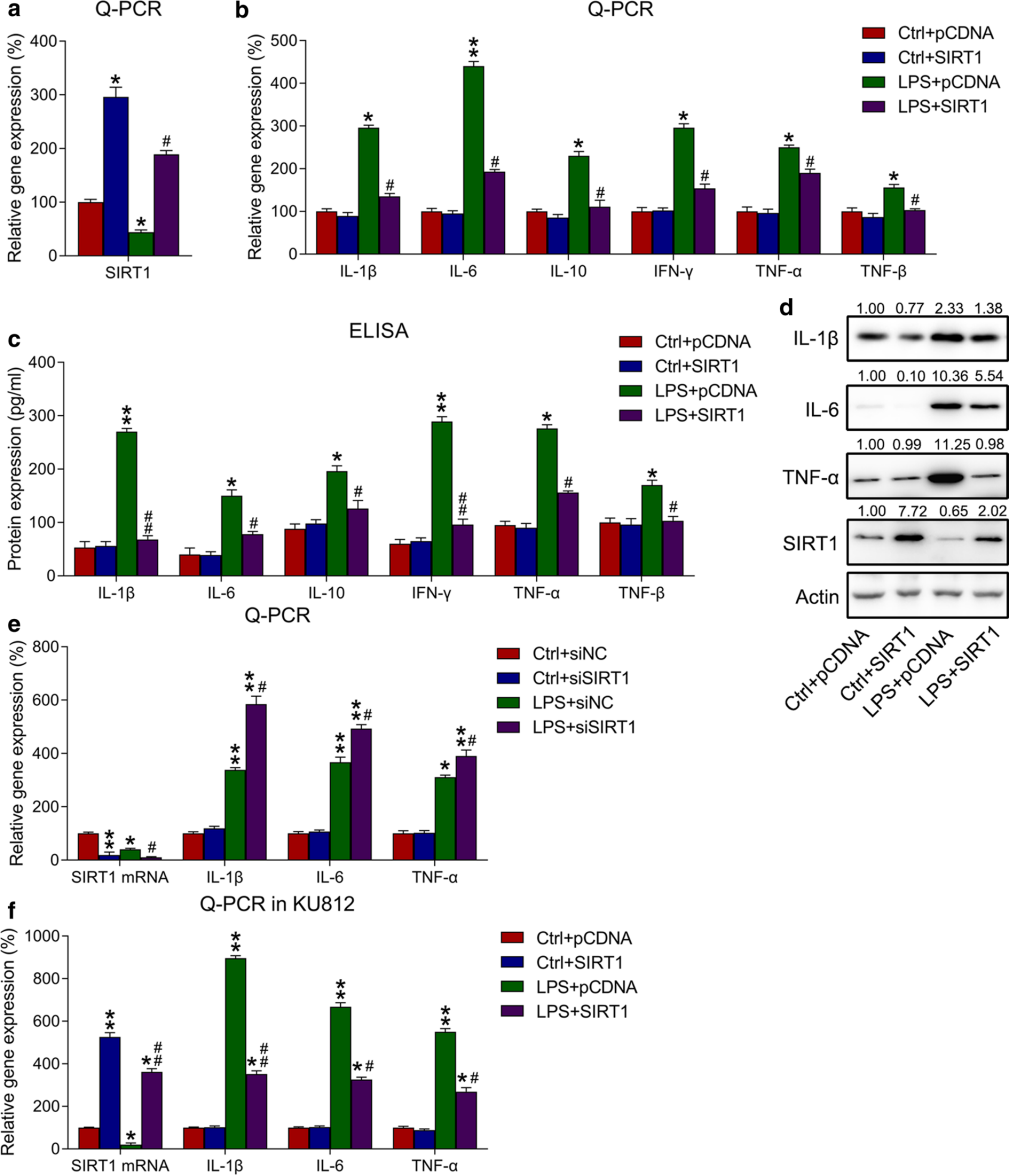
Influence of SIRT1 overexpression on inflammatory responses in k562 cells. **a** k562 cells were transfected with SIRT6-expressing plasmid/empty plasmid, followed by exposure to 10 μg/mL of LPS for 4 h or not. qPCR showed the mRNA expression of SIRT1 in k562 cells with different transfection and incubation. **b**, **c** Influence of LPS treatment and SIRT1 overexpression on the mRNA and protein expressions of inflammatory regulators determined using qPCR and ELISA. In qPCR, gene expression in each group was first normalized to the GAPDH gene, and then normalized to the data of the control group. **d** WB was performed to assess the protein expressions of SIRT1, IL-1β, IL-6, and TNF-α. **e** k562 cells were transfected with siRNA-NC or siRNA-SIRT1, followed by exposure to 10 μg/mL of LPS for 4 h or not. Influence of LPS treatment and SIRT1 silencing on the mRNA expressions of inflammatory regulators performed via qPCR. **f** KU812 cells were transfected with SIRT6-expressing plasmid/empty plasmid, followed by exposure to 10 μg/mL of LPS for 4 h or not. Influence of LPS treatment and SIRT1 overexpression on the mRNA expressions of inflammatory regulators in KU812 cells performed via qPCR. Data are expressed as mean ± standard deviations. *P < 0.05, **P < 0.01 and ^#^P < 0.05, ^##^P < 0.01 relative to the control + pCDNA/siNC group and LPS + pCDNA/siNC group, respectively

To determine the influence of SIRT1 on inflammatory factor production in k562 cells, these cells were first transfected with SIRT1-expressing plasmid or empty plasmid and then treated with either LPS or the control formulations. SIRT1 expression was detected via WB. Following SIRT1 overexpression, SIRT1 mRNA and protein levels were dramatically increased, even in the LPS group (Fig. 3a, d). Subsequently, qPCR, ELISA and WB were used to detect the mRNA and protein levels of inflammatory cytokines, respectively. Ultimately, SIRT1 overexpression was found to significantly reduce the mRNA and protein levels of cytokines in LPS-treated k562 cells (Fig. 3b–d). To determine the effects of SIRT1 silencing on LPS-induced inflammation, k562 cells were transfected with siRNA-NC or siRNA-SIRT1, and then either treated or not treated with LPS. qPCR was performed to examine the expression of IL-1β, IL-6, TNF-α, and SIRT1 mRNA expressions. The data showed that SIRT1 silencing did not affect cytokines production in the group not treated with LPS, however, SIRT1 knockout augmented the expression of LPS-induced cytokines of k562 cells (Fig. 3e). Similar observations for IL-1β, IL-6, and TNF-α mRNA levels in KU812 cells with SIRT1 overexpression were obtained using the qPCR data (Fig. 3f), which revealed that SIRT1 overexpression inhibited IL-1β, IL-6, and TNF-α expressions at the mRNA level in KU812 cells.

### Effects of SIRT1 overexpression on the inflammation-associated TLR4–NFκB–ROS axis

**Fig. 4:**
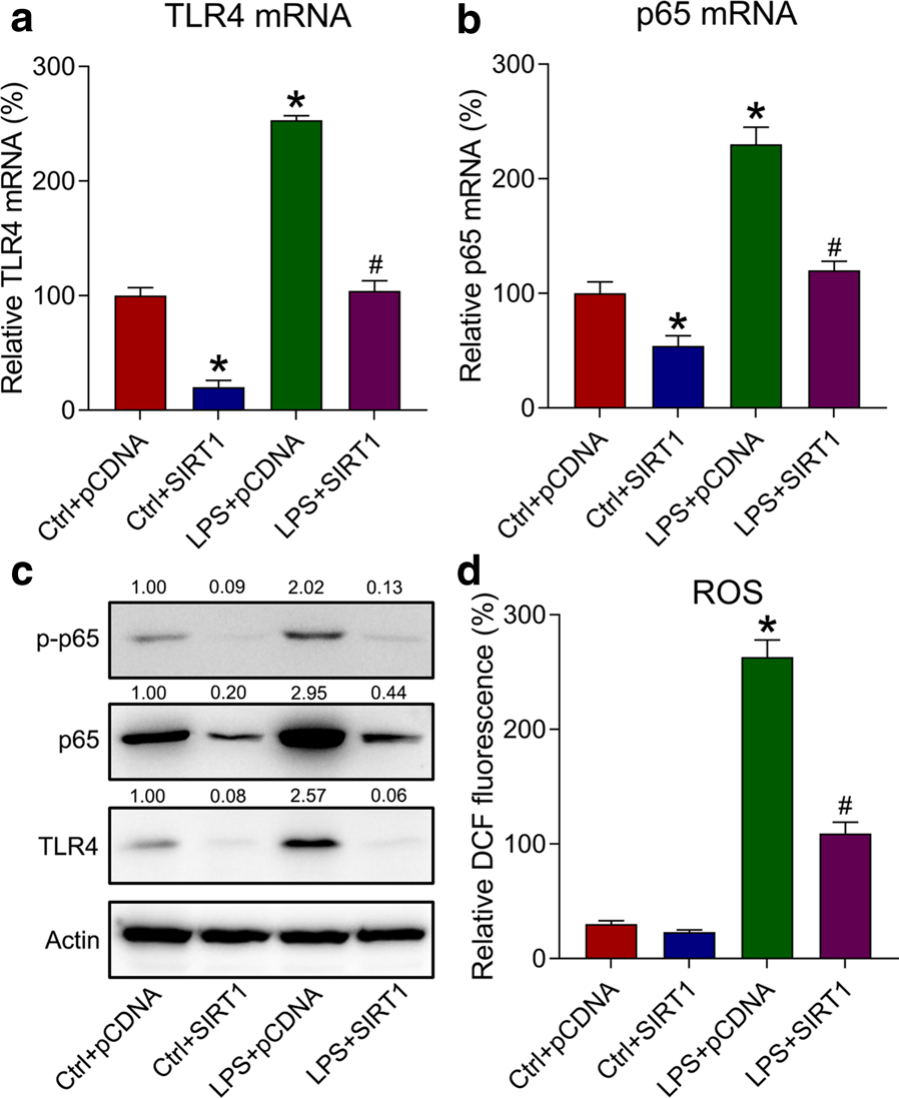
Role of SIRT1 overexpression in activating the TLR4–NFκB–ROS axis in LPS-treated k562 cells. Here, k562 cells were transfected with SIRT6-expressing plasmid/empty plasmid, followed by exposure to 10 μg/mL of LPS or not for 4 h. **a**, **b** The mRNA expressions of TLR4 and p65 were detected by qPCR. Gene expression in each group was first normalized to the GAPDH gene, and then normalized to the data of the LPS-pCDNA group. **c** The influence of SIRT1 overexpression on TLR4, p65, and p65 phosphorylation was assessed using WB. **d** ROS production reflected by DCF fluorescence intensity was determined in LPS-treated cells. Data are expressed as mean ± standard deviation. *P < 0.05 relative to the control + pCDNA group; ^#^P < 0.05 relative to the LPS + pCDNA group

The TLR4**–**NFκB–ROS signaling axis has been highly associated with the onset of inflammation [16, 17]; therefore, WB and qPCR analyses were utilized to assess the involvement of this axis in LPS-induced inflammation and its relation with SIRT1. Cells transfected with SIRT1-expressing plasmid were either treated or not treated with LPS, subsequently, we detected the expression of vital regulators of the TLR4–NFκB–ROS axis. Both the mRNA and protein expressions of TLR4 and p65 were upregulated due to LPS treatment, but downregulated after SIRT1 overexpression (Fig. 4c). Meanwhile, p65 phosphorylation was also decreased due to SIRT1 overexpression. To investigate whether ROS production was also reduced by SIRT1 overexpression, ROS production was determined using DCFH-DA. LPS administration resulted in the a robust ROS production in k562 cells, whereas SIRT1 overexpression led to reduced ROS generation (Fig. 4d). These observations collectively indicate that SIRT1 mitigated the inflammation caused by LPS through modulating the TLR4–NFκB–ROS axis.

### ROS production is responsible for LPS-induced inflammation in k562 cells

**Fig. 5:**
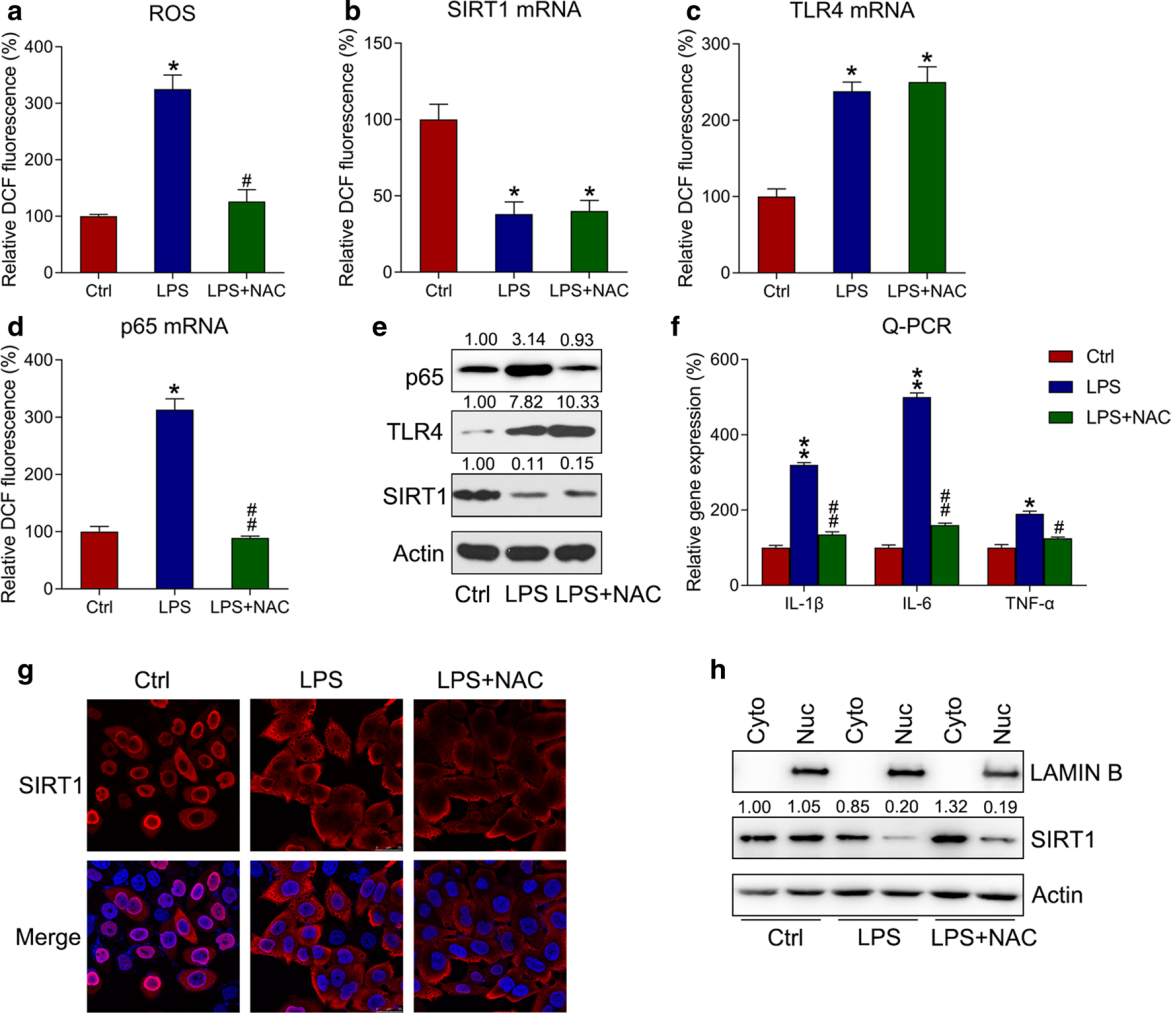
ROS generation is responsible for SIRT1-mediated inflammation in k562 cells. Here, k562 cells were subjected to 5 μM of NAC and 10 μg/mL of LPS for 4 h. **a** ROS production in k562 cells was reflected by DCF fluorescence intensity. **b**–**d** qPCR was performed to determine the mRNA levels of SIRT1, TLR4, and p65 in k562 cells. **e** WB was performed to determine the protein levels of SIRT1, TLR4, and p65 in k562 cells. **f** qPCR was performed to determine the mRNA levels of IL-1β, IL-6, and TNF-α in k562 cells. Gene expression in each group was first normalized to the GAPDH gene, and then normalized to the data of the control group. **g** SIRT1 subcellular localization in LPS-treated k562 cells detected viaIFA. SIRT1 was stained to red through anti-SIRT1 antibody (red), whereas nuclear DNA was counterstained blue through DAPI (blue). The merged image displays the nuclear localization of SIRT1. **h** Cell fractionation assay showed the location of SIRT1 in nuclear and cytoplasmic fractions. Data are expressed as mean ± standard deviation. *P < 0.05, **P < 0.01 and ^#^P < 0.05, ^##^P < 0.01 relative to the control and LPS groups, respectively

We hypothesized that reducing ROS production could result in the SIRT1-mediated inactivation of the TLR4–NFκB–ROS axis and the alleviation of inflammation. To explore this, k562 cells were cotreated with 5 μM of NAC, a ROS scavenger [18], and LPS for 4 h. LPS-induced ROS generation was clearly reduced with NAC treatment (Fig. 5a). qPCR and WB were used to detect the mRNA and protein expressions respectively, of SIRT1, TLR4, and p65 in response to NAC treatment. NAC treatment did not significantly alter the mRNA expressions of SIRT1 and TLR4 (Fig. 5b, c, e), whereas it downregulated the mRNA expression of p65 (Fig. 5d, e), suggesting the existence of cross-talking between p65. qPCR was used to detect IL-1β, IL-6, and TNF-αmRNA levels, and it was found that NAC pretreatment dramatically reduced inflammatory cytokine levels triggered by LPS (Fig. 5f); this suggested that SIRT1 could participate in LPS-triggered cytokine generation by modulating ROS production.

## Discussion

The present study results showed that LPS-triggered inflammation accompanied reduced SIRT1 expression in k562 cells. SIRT1 overexpression also could decrease LPS-induced inflammatory cytokine production and obviously reduce inflammation-relevant TLR4 sensors and the activation of downstream NFκB p65. In addition, ROS generation via TLR4–NFκB p65 activation was also reduced by SIRT1 overexpression. The administration of the ROS inhibitor NAC to LPS-treated k562 cells also led to amelioration of inflammation. The study demonstrated that SIRT1 inhibited LPS-induced inflammatory reactions in k562 cells through the abrogation of the TLR4–NFκB–ROS axis.

CML is a clonal hematological malignancy that arises resulting from BCR–ABL-transformed HSCs [1]. SIRT1 expression is significantly increased in CML LSCs compared with that in their normal counterparts [19]. BCR–ABL activates SIRT1 through kinase-dependent STAT5 signaling. However, BCR–ABL kinase inhibition or STAT5 knockdown can only partially reduce SIRT1 expression, suggesting that other kinase-independent mechanisms are responsible for increased SIRT1 activity in CML [19]. The genetic loss of SIRT1 can also increase p53 acetylation in CML LSCs [20]. LPS treatment dramatically elevated the expression of proinflammatory modulators and cytokines at protein and mRNA levels in the present study. Meanwhile, the SIRT1 expression decreased after LPS treatment. Previous studies have demonstrated that LPS causes Sirt1 downregulation in endothelial cells [21–23]. Furthermore, one study has demonstrated that LPS treatment decreases SIRT1 stability by inactivating JNK during the inflammatory responses of LPS-activated macrophages [24]. To investigate the function of SIRT1 in CML k562 cells, in the present study, SIRT1 was overexpressed using transfection with pcDNA3-SIRT1. Ultimately, SIRT1 overexpression reduced p65 phosphorylation and activation in CML k562 cells, reducing the cellular inflammatory response.

TLRs play an essential roles in nonspecific immunity, identify numerous exogenetic PAMPs and pathogens and initiate endogenic immune reactions [25, 26]. TLR4, a downstream MAPK signal activator and PRR-binding protein, is reportedly inactivated by SIRT1 overexpression [27]. Simultaneously, its downstream NFκB pathway in SIRT6-overexpressing cells is likewise inhibited [28]. After activation, TLRs exhibit the key effect of a strong defense against invasive pathogens. LPS may induce TLR4 to activate the downstream NFκB signaling pathway, which can elicit cytokine production and the expressions of various inflammation-relevant genes [16, 27, 28]. In the present study, LPS could activate the TLR4 signaling pathway, whereas SIRT1 overexpression exhibited the contrary effect. We also observed that SIRT1 could repress NFκB phosphorylation. These observations indicated that SIRT1 could modulate LPS-triggered inflammation by abrogating TLR4–NFκB.

Increased levels of ROS have been reported to be deleterious to cells due to the oxidative stress, resulting from the interactions of ROS with nucleic acids, lipids, and proteins [29]; ROS also play essential parts in inflammatory reactions by activating NFκB, especially in immune cells. For instance, cyclooxygenase-2, a well-known NFκB target, can transform arachidonic acids to PGH2 [30]. Increased ROS production has been demonstrated as one of the defining characteristics of CML [31, 32]. Reducing ROS levels may restrain the CML development, given the high degree of oxidative stress typical of hematological malignancies [33]. The present study revealed that LPS treatment increased ROS production, despite the inhibition of this production by SIRT1 overexpression. Furthermore, NAC treatment reduced LPS-induced inflammatory cytokine levels. These findings indicated that ROS production was involved in SIRT1-mediated inflammation triggered by LPS. One limitation of our study is that we could not determine the type of ROS was involved in the SIRT1-mediated inflammation in LPS-treated CML cells, due to the nonspecific property of DCFH-DA assay and ROS scavenger NAC. The type of ROS species will be determined in our future study.

## Conclusions

Our data suggest that SIRT1 exhibits a protective effect in CML k562 cells against LPS-triggered inflammation. SIRT1 ameliorates the inflammation elicited by LPS in k562 and KU812 cells, probably by inactivating the TLR4–NFκB–ROS axis. These results may help find a new treatment method for inflammation in CML cells.

## Data Availability

All data generated or analyzed during this study are included in this published article.

## Abbreviations

CML: Chronic myelogenous leukemia
MPN: Myeloproliferative neoplasm
SIRT1: Sirtuin 1
LPS: Lipopolysaccharide
IL: Interleukin
IFN: Interferon
IFA: Immunofluorescence assay
PBS: Phosphate-buffered saline
mRNA: Messenger RNA
TNF: Tumor necrosis factor
TLR4: Toll-like receptor 4
NFκB: Nuclear factor κ B
ROS: Reactive oxygen species
TKIs: Tyrosine kinase inhibitors
SIRTs: Sirtuins
NAD: Nicotinamide adenine dinucleotide
WB: Western blot
qPCR: Quantitative polymerase chain reaction
mRNA: Messenger RNA
ELISA: Enzyme-linked immunosorbent assay
